# Integrated Analysis of Hub Genes and miRNAs in Dilated Cardiomyopathy

**DOI:** 10.1155/2020/8925420

**Published:** 2020-09-15

**Authors:** Kai Huang, Shuyan Wen, Jiechun Huang, Fangrui Wang, Liewen Pang, Yiqing Wang, Xiaotian Sun

**Affiliations:** Department of Cardiothoracic Surgery, Huashan Hospital of Fudan University, Shanghai, China

## Abstract

**Purpose:**

The aim of this study is to identify hub genes and miRNAs by the miRNA-mRNA interaction network in dilated cardiomyopathy (DCM) disease.

**Methods:**

The differentially expressed miRNAs (DEMis) and mRNAs (DEMs) were selected using data of DCM patients downloaded from the GEO database (GSE112556 and GSE3585). Gene Ontology (GO) pathway analysis and transcription factor enrichment analysis were used for selecting DEMis, and the target mRNAs of DEMis were filtered by using miRDB, miRTarBase, and TargetScan. Cytoscape software was used to visualize the network between miRNAs and mRNAs and calculate the hub genes. GO and Kyoto Encyclopedia of Genes and Genomes (KEGG) pathway analyses were used to analyze the mRNAs in the regulatory network.

**Results:**

A total of 9 DEMis and 281 DEMs were selected, from which we reconstructed the miRNA-mRNA network consisting of 7 miRNAs and 51 mRNAs. The top 10 nodes, miR-144-3p, miR-363-3p, miR-9-3p, miR-21-3p, miR-144-5p, miR-338-3p, ID4 (inhibitor of DNA binding/differentiation 4), miR-770-5p, PIK3R1 (p85*α* regulatory subunit of phosphoinositide 3-kinase (PI3K)), and FN1 (fibronectin 1), were identified as important regulators.

**Conclusions:**

The study uncovered several important hub genes and miRNAs involved in the pathogenesis of DCM, among which, the miR-144-3p/FN1 and miR-9-3p/FN1 pathways may play an important role in myocardial fibrosis, which can help identify the etiology of DCM, and provide potential therapeutic targets.

## 1. Introduction

Dilated cardiomyopathy (DCM) is a primary cardiac disease involving genetic or postinflammatory etiology [1]. Left ventricular (LV) or both ventricular dilatation and systolic dysfunction are the main physical signs, coupling with cardiac remodeling and fibrosis. In clinical practice, these manifestations cannot be easily interpreted by volume overload following hypertension or valve disease or by coronary artery disease (CAD) causing global systolic dysfunction [2], despite the typical phenotype, the diversity of DCM pathophysiology, the adverse consequences of myocardial biopsy, and the lack of marker protein for making a definite diagnosis call for innovation to identify pathophysiology mechanism, which can provide clinical decision support [3].

In recent years, the microRNA (miRNA) has gained more and more attention from researchers in cardiovascular disease study. Several reports have revealed the critical roles of miRNA and targeted message RNA (mRNA) in the development of various diseases, including cardiovascular disorders [4, 5]. By using a rodent cardiomyopathy model induced by doxorubicin, Tao et al. revealed that miR-144-3p and miR-451a were downregulating and miR-21-5p was upregulating [6]. Gioffre et al. also proved a close connection between miR-34a-5p and miR-451a to DOX-induced cardiomyopathy [7]. Chen et al. suggested that miR-223-3p regulates immune tolerance of dendritic cells in autoimmune myocarditis [8]. mRNAs also serve as a diagnostic biomarker for DCM [9, 10] and can value the progression of myocardial fibrosis and ventricular remodeling in heart failure [11–13]. However, few researches have been done to clarify the miRNA-mRNA regulatory network in DCM disease.

In this study, we retrieved microarray data of miRNAs and mRNAs in heart chamber samples from DCM patients for heart transplant and compared them with control data of donor hearts. After screening the differential expression of miRNAs and mRNAs in the two groups, we reconstructed the miRNA-mRNA network according to the miRNA sponge theory [14]. The study discriminated human DCM-related miRNAs with high credibility and provided a novel approach to identify pathological mechanisms and potential targets for DCM.

## 2. Materials and Methods

### 2.1. Data Download and Screening Strategy

Microarray data sets of DCM were downloaded from Gene Expression Omnibus (https://www.ncbi.nlm.nih.gov/geoprofiles). The miRNA expression profile data GSE112556 [6] performed on Platform GPL18402 contained myocardial tissues from three healthy people and three dilated cardiomyopathy patients. The gene/mRNA expression profile GSE3585 [15] was performed on Platform GPL96, with biopsy from seven DCM patients and five donor hearts.

We used the limma package in R studio to get the differentially expressed miRNAs (DEMis) and mRNAs (DEMs) between the DCM cases and healthy controls. Bayesian methods corrected the batch effect. If more than one probe mapped into the same gene, the average expression value was used to equal the expression value of that gene. The *t*-test was applied to filter the differentially expressed genes. The DEMis and DEMs were screened by the *P* values < 0.05 and log FC > average (log FC) + 2∗SD (log FC) [16]. To show the differential expression of DEMis and DEMs in different samples, volcano maps and heat maps were drawn by applying the plot and pheatmap packages in the R studio.

### 2.2. GO Enrichment Analysis for the Targets of Transcription Factors

The DEMis were uploaded to FunRich (3.1.3), which is a commonly used tool for GO functional enrichment analysis of enriched targets (genes/mRNAs) of transcription factor pathways. GO enrichment analysis was also applied to develop the interaction network analysis between miRNAs, gene/mRNA, and transcription factors (already integrated the explored information of miRNA and potentially targeted gene/mRNAs) [17–19]. The enriched targets of DEMis and involved pathways were explored by previous methods [20].

### 2.3. Prediction of the Targeted mRNAs of DEMis

The miRDB (http://www.mirdb.org), miRTarBase (http://mirtarbase.mbc.nctu.edu.tw), and TargetScan (http://www.targetscan.org) databases were used to predict the targeted mRNAs of DEMis gained above. After that, the predicted mRNAs of DEMis were further filtered by matching the DEMs selected before, and then we got the DEMi-DEM pairs.

### 2.4. Construction of the miRNA-mRNA Regulatory Network

The miRNA-mRNA network was constructed by putting all the DEMi-DEM pairs selected above together, and Cytoscape software (version 3.7.2) was used to visualize it at the same time. All the node degrees, closeness, and betweenness of the regulatory network were calculated simultaneously.

### 2.5. GO and KEGG Enrichment Analyses on mRNAs in the Network

We used enrichplot and ggplot2 packages in the R studio to perform Gene Ontology (GO) and Kyoto Encyclopedia of Genes and Genomes (KEGG) pathway analyses on mRNAs in the network. *P* value < 0.05 was considered statistically significant.

### 2.6. Statistical Analysis

The significant differences between the two groups were analyzed by Student's *t*-test. To further control the error rate, the Benjamini and Hochberg method was used to calculate the adjusted *P* value. A value of *P* < 0.05 or adjusted *P* value < 0.05 was considered to be significant. All authors had full access to and take full responsibility for the integrity of the data.

## 3. Results

### 3.1. The DEMi Screening Results in DCM

**Figure 1:**
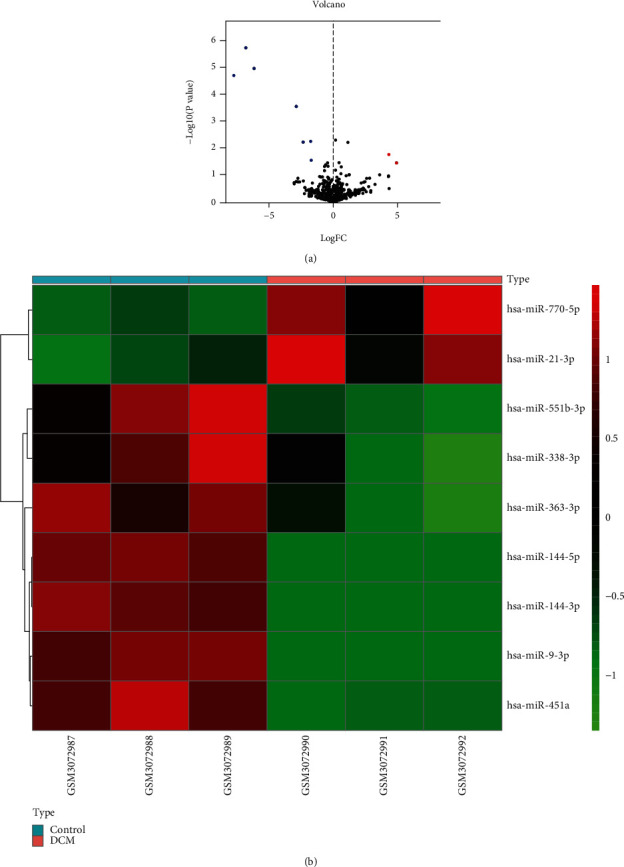
Volcano map of DEMis. Red spots represent upregulated genes; green spots represent downregulated genes (a). Heat map of DEMis. The left three samples were from the control group, and the right three samples were from the DCM group. Red color: high expression; blue color: low expression (b).

According to the filtering criterion described before, the cutoff value for log FC of miRNAs was 1.5. We identified nine dysregulated DEMis, including 2 upregulated miRNAs: miR-770-5p and miR-21-3p, and 7 downregulated miRNAs: miR-144-3p, miR-144-5p, miR-9-3p, miR-451a, miR-551-3p, miR-363-3p, and miR-338-3p. All the nine DEMis are presented in Table 1. The distribution of differential miRNA expressions between DCM and healthy controls was intuitively illustrated by the volcano map on the correlation of −log10 (*P* value) and log (FC) (Figure 1(a)). The heat map was also drawn to show the differences between DCM and healthy groups (Figure 1(b)).

### 3.2. Transcription Factor Enrichment and GO Enrichment Analyses

**Figure 2:**
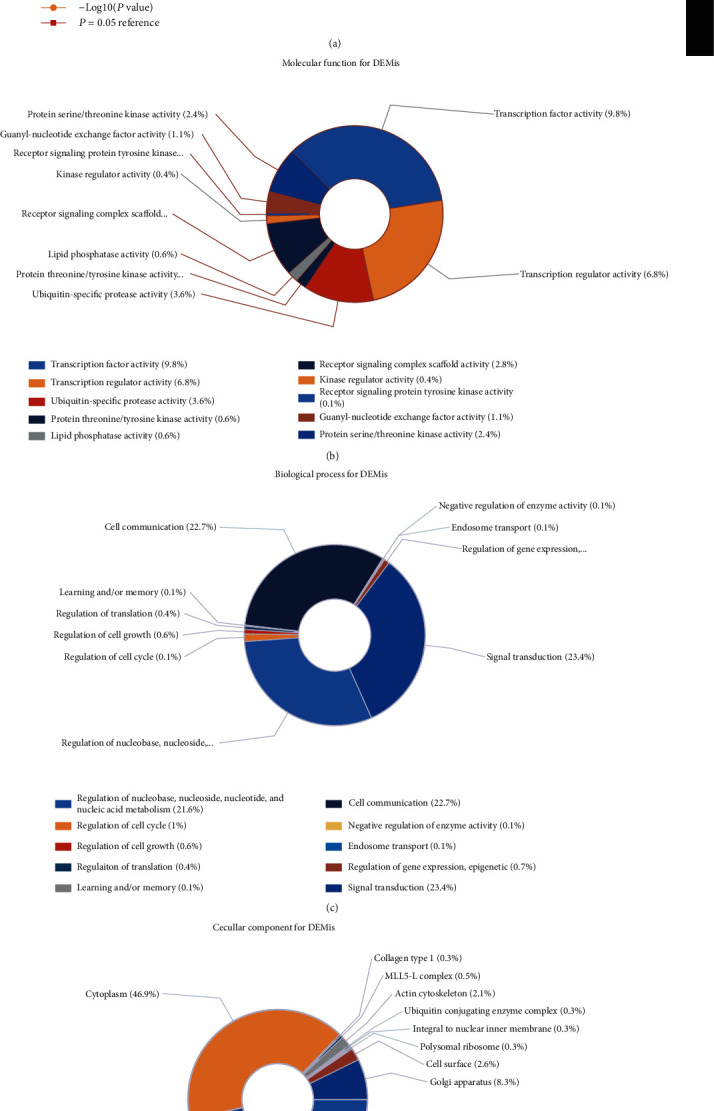
The transcription factor enrichment for DEMis (a); genes/mRNAs involved in molecular function terms for DEMis (b); genes/mRNAs involved in biological process terms for DEMis (c); genes/mRNAs involved in cellular component terms for DEMis (d).

A total of 4297 genes were mapped into 55 transcription factors. By exploring the enrichment of targets of transcription factors, we filtered the top 10 transcription factors which had strong closeness to miRNAs, including SP1, EGR1, SP4, LHX3, CUX1, POU2F1, HOXD8, MEF2A, HOXA9, and NKX6-1 (Figure 2(a)), suggesting these transcription factors being in regulatory relationships with DEMis.

As to the molecular function (MF) terms by GO enrichment analysis, most of the genes were involved in transcription factor activity, transcription regulator activity, ubiquitin-specific protease activity, lipid phosphatase activity, and protein threonine/tyrosine kinase activity (Figure 2(b)). GO enrichment analysis also revealed that the top 5 biological progress (BP) terms with the most enriched targets of DEMis include regulation of nucleobase, nucleoside, nucleotide, and nucleic acid metabolism; regulation of cell cycle, cell growth, translation, learning, and memory; cell communication and negative regulation of enzyme activity; endosome transport; and regulation of gene expression, epigenetic, and signal transduction (Figure 2(c)). The top 5 of the cellular component (CC) terms with the most enriched targets of DEMis were proved to be the nucleus cytoplasm, collagen type I, MLL5-L complex, actin cytoskeleton, and ubiquitin-conjugating enzyme complex (Figure 2(d)).

### 3.3. Results of the DEM Screening in DCM

**Figure 3:**
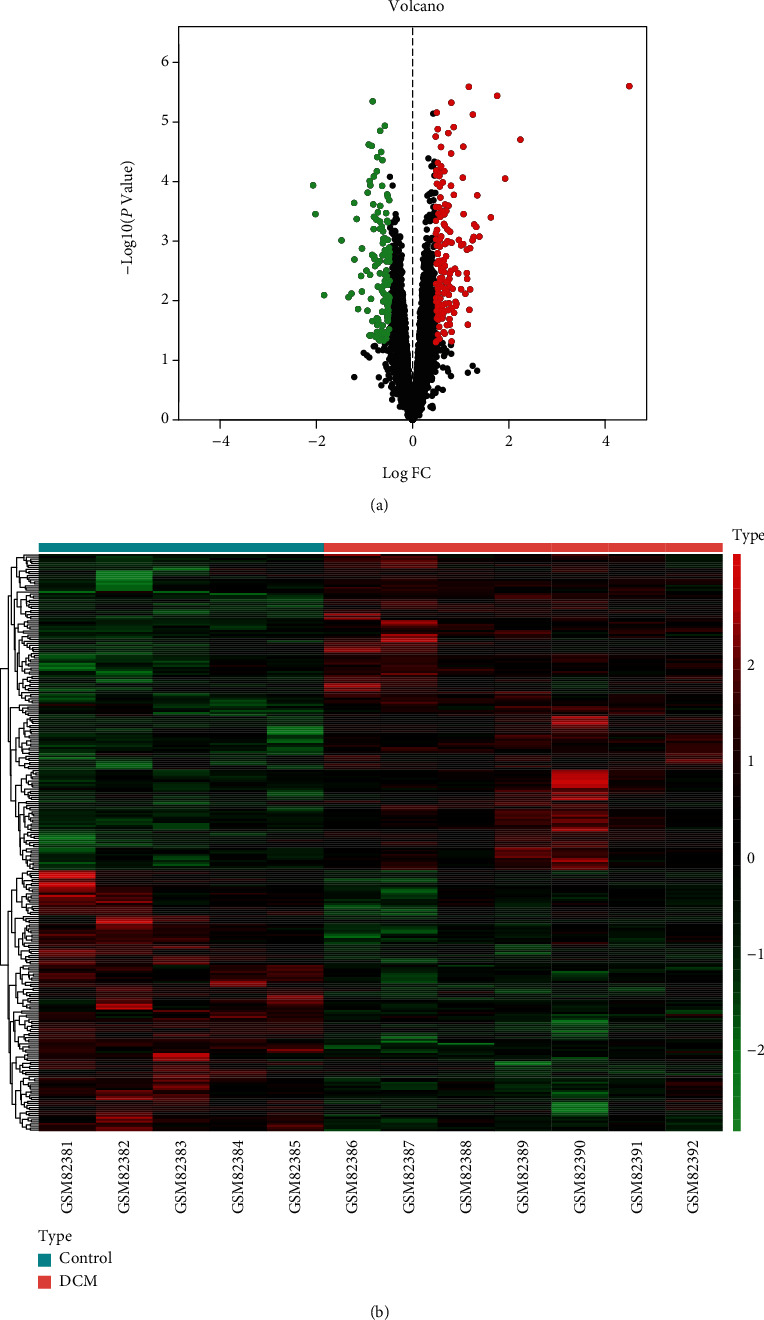
Volcano map of DEMs. Red spots represent upregulated genes, and green spots represent downregulated genes (a). Heat map of DEMs. The green color represents low expression, and the red color represents high expression (b).

In this study, the expression levels of mRNAs from 7 DCM patients and 5 healthy controls were explored. The threshold value for log (FC) of mRNAs is 0.47; 154 (54.8%) upregulated and 127 (45.2%) downregulated mRNAs were confirmed. The top 20 of upregulated and downregulated genes are shown in Table 2. Both the volcano plot and the heat map are shown in Figures 3(a) and 3(b), respectively.

### 3.4. Reconstruction of the miRNA-mRNA Network in DCM

**Figure 4:**
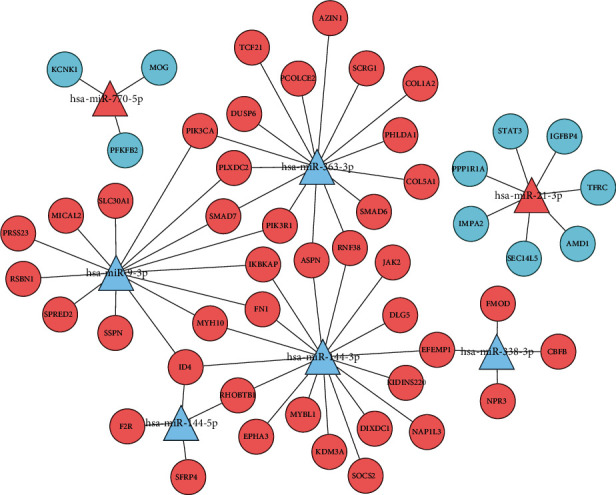
The miRNA-mRNA network. The upregulated genes were exhibited by the red color, while the blue color exhibited the downregulated genes. MicroRNAs are represented by a circular shape, and miRNAs are represented by a triangle shape.

As showed in Figure 4, a miRNA-mRNA regulatory network bearing 51 mRNAs and 7 miRNAs was constructed to further exhibit the interaction between DEMis and DEMs, which is beneficial to understand the role of miRNAs in DCM. The parameters of degree, closeness, and betweenness in the network were calculated by the plugin cytoHubba in Cytoscape (version 3.7.2). The top 10 nodes, including miR-144-3p, miR-363-3p, miR-9-3p, miR-21-3p, miR-144-5p, miR-338-3p, ID4, miR-770-5p, PIK3R1, and FN1, could be selected as hub nodes (Table 3). Three miRNAs (miR-144-3p, miR-363-3p, and miR-9-3p) were considered to have the most node degrees, suggesting which might play critical roles in the genesis and development of DCM as the key miRNAs. In the top 10 nodes, ID4 were the target mRNAs of miR-144-3p and miR-9-3p; PIK3R1 were the target mRNAs of miR-363-3pa and miR-9-3p; and FN1 were the target mRNAs of miR-144-3p and miR-9-3p. These 6 pathways could be involved in the development of DCM.

### 3.5. Functional Enrichment Analysis of mRNAs in the Regulatory Network

**Figure 5:**
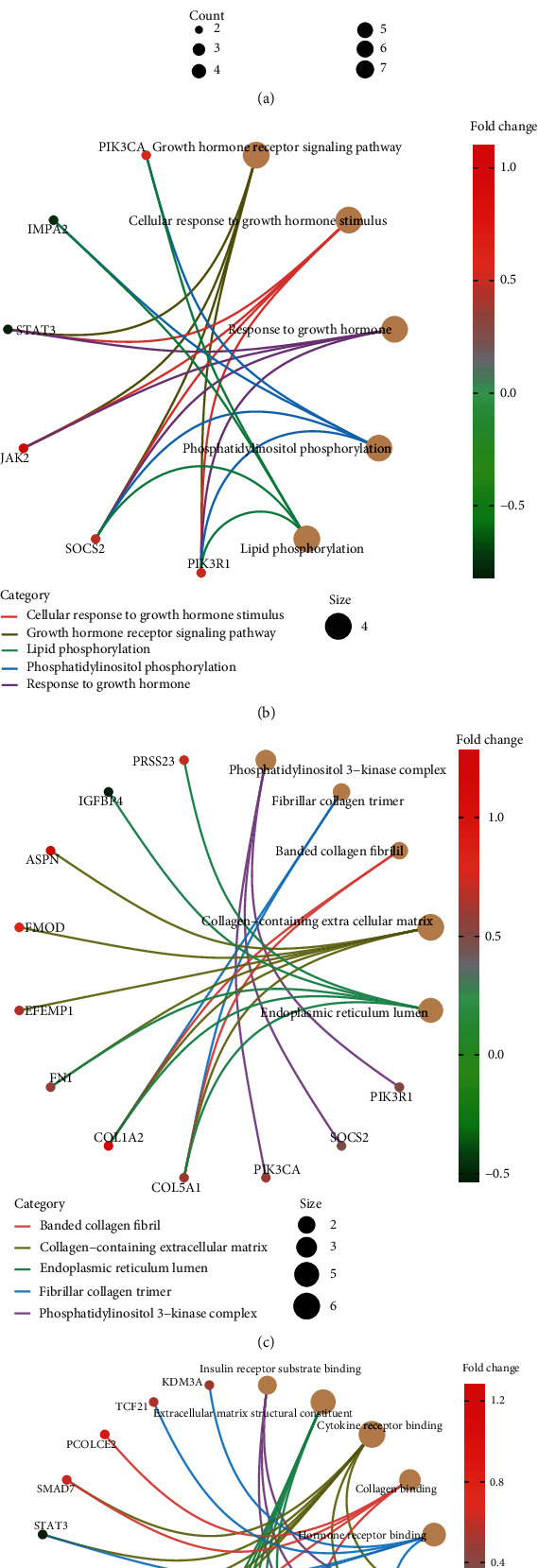
The top 5 enriched GO biological process terms of DEMs in DCM (a); the relationship between enriched mRNAs in BP pathways (b); CC pathways (c); MF pathways (d).

**Figure 6:**
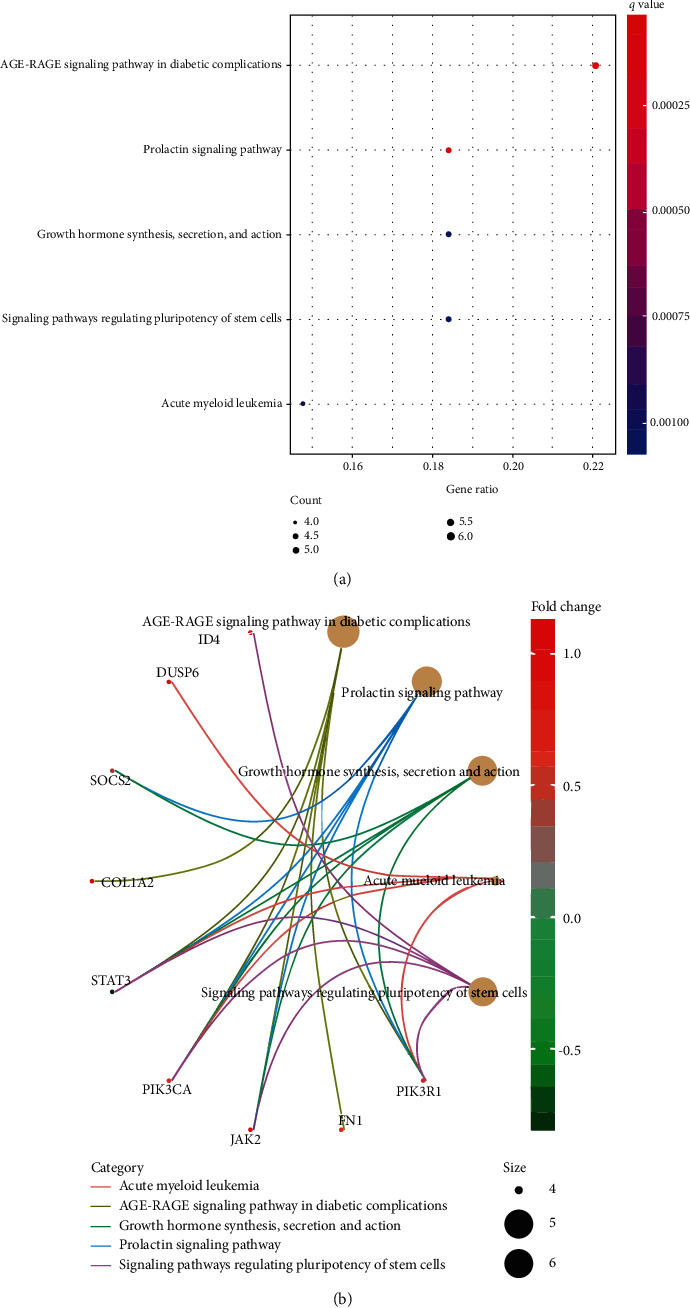
The KEGG pathway enrichment analysis of mRNAs in the network (a). The relationship between enriched mRNAs in KEGG pathways (b).

GO analysis of the mRNAs in the regulatory network showed that BP terms were significantly enriched in the growth hormone receptor signaling pathway, cellular response to growth hormone stimulus, response to growth hormone, phosphatidylinositol phosphorylation, and lipid phosphorylation. The cell component (CC) terms were enriched in phosphatidylinositol 3-kinase complex, collagen trimer, banded collagen fibril, collagen-containing extracellular matrix, endoplasmic reticulum lumen, and complex of collagen trimers. The molecular function terms included insulin receptor substrate binding, extracellular matrix structural constituent, cytokine receptor binding, collagen binding, and hormone receptor binding (Figure 5(a)). The relationships between mRNAs and enriched pathways are also shown in Figures 5(b)–5(d). The most important 5 pathways by KEGG are also shown in Figure 6(a). Including the AGE-RAGE signaling pathway in diabetic complications, the prolactin signaling pathway, growth hormone synthesis and secretion, and signaling pathways regulating pluripotency of stem cells were involved in the pathological development of DCM. The relationships between mRNAs and enriched KEGG pathways are shown in Figure 6(b).

## 4. Discussion

DCM is one of the main reasons for sudden death and heart failure globally, with an estimated prevalence of 1 in 2500 people and an incidence of 7 in 100,000 people annually [21]. However, the etiology of DCM is still unclear. Infection, noninfectious inflammation, poisoning, endocrine and metabolic disorders, familial inheritance, and trauma are all included. A major understanding of cardiomyopathies stemming from the frequent identification of underlying genetic causes involves mutations in myocardial proteins, cell-cell communication, and the cytoskeleton. These, in turn, lead to abnormal contraction and relaxation or dysregulated ion transportation across cell membranes. As a structural heart disease, DCM causes the dilatation of the heart ventricle and the damage of systolic function, leading to heart failure as the final stage [22]. Despite some progress in the treatment and diagnosis, the prognosis of DCM patients remains poor at the current stage.

The pathological process of DCM is dominated by the enlargement of the cardiac chamber, following visible ventricular dilatation, a variable and thin ventricular wall, the formation of a fibrous scar, and often with the mural thrombus. Histologically, nonspecific hypertrophy and degeneration of cardiomyocytes and especially different degrees of fibrosis were mixed together. A variety of inflammatory cell infiltration could also be observed in the inflammatory process. The interactions of miRNAs and mRNAs are involved in almost all biological processes. Various studies proved that miRNAs play an important role in the process of pathology relating to DCM. The impact of miR-1 in cardiac hypertrophy targeting was clearly described [23]; the miR-133a level was observed to be correlated with macrophage infiltration, cardiac injury, and clinical outcome in DCM patients [24]. Duisters et al. found that miR-133 and miR-30 could downregulate the connective tissue growth factor and the key fibrosis-related protein, playing an important role in the structural changes in the extracellular matrix of the myocardium [25].

In this study, we reconstructed the miRNA-mRNA regulatory network composed of 7 miRNAs and 51 mRNAs. We also calculated the degree, closeness, and betweenness of genes in the network. The top 10 nodes, including miR-144-3p, miR-363-3p, miR-9-3p, miR-21-3p, miR-144-5p, miR-338-3p, miR-770-5p, ID4, PIK3R1, and FN1, were selected as hub nodes in the development of DCM. In the regulatory network, miR-144-5p, miR-144-3p, miR-9-3p, miR-363-3p, and miR-338-3p were downregulated in DCM samples compared with the healthy controls. Meanwhile, miR-770-5p and miR-21-3p were upregulated in DCM samples.

In order to better understand the mechanisms of target mRNAs in DEMis, the present study filtered out possible transcription factors. Specificity Protein 1 (SP1) is the most common transcription factor. SP1 was an important regulator in the development of neonatal cardiomyocytes. In this work, we filtered miR-144 as a hub node in the regulation of DCM. miR-144/451 was found to be tightly clustered and evolutionally conserved. By targeting the CUGBP2-COX2 signaling pathway, the upregulated miR-144/451 cluster can protect the heart from hypoxic stress [26]. Yuan et al. found that in the myocardium infarct zone, the expression level of miR-144-3p was increased, and the decreased expression levels of miR-144-3p were accompanied with the decreased mRNA and related protein levels of the fibrosis-related genes [27]. Similarly, the expression level of miR-9 was upregulated in H9C2 cells subject to hypoxia, and the hypoxia-induced cardiomyocyte apoptosis was inhabited by the miR-9 knockdown procedure [28]. The expression level of miR-9-5p decreased in the ischemic myocardium [29]. Jin et al. established the rodent myocardial infarction model and proved that compared with the sham group, the rats in the miR-9 group had substantially decreased type I and type III collagen, suggesting that miR-9 can alleviate the myocardium fibrosis process in cardiomyopathy [30]. According to existing literatures, miR-144 and miR-9 might play an important role in the hypoxic stress, apoptosis, and fibrosis process in DCM.

Meng et al. proved that inhibition of miR-363 could protect the cardiomyocyte against hypoxia-induced apoptosis through the regulation of Notch signaling [31]. miR-338 was involved in the development of cardiac hypertrophy [32]. However, few studies revealed the role of miR-338 in DCM. miR-770 exerts its role more in cancer [33] and diabetes [34], suggesting that it has potential to be an entirely new target in DCM. At the same time, miR-21 has been improved to participate in various pathophysiological mechanisms of cardiovascular diseases, such as inflammatory infiltration [35], apoptosis, oxidative stress [36], and cardiac fibrosis [37].

PIK3R1, ID4, and FN1 were selected as the key genes in the miRNA-mRNA network. The mutation of PIK3R1 relating to insulin resistance can affect both cardiac metabolism and contractile function. Young et al. and Chen et al. [38, 39] found that PIK3R1 was involved in circadian rhythms, glucose utilization, physiologic, metabolic stress, and cardiac contractile function. In our study, PIK3R1 is the target gene of miR-9 and miR-363, which may broaden our understanding of the relationship between DCM and metabolism. ID4 encodes a member of the inhibitor of the DNA binding (ID) protein family, which also takes part in the mammalian circadian system [40]. FN1 encodes fibronectin, which plays essential roles in cell adhesion and migration, including the formation of the embryo, healing, blood clotting response, and host defense. The role of FN1 in DCM focuses on extracellular cellular matrix (ECM) remodeling [41]. As the target gene of miR-144 and miR-9, it can be concluded that the miR-144/FN1 and miR-9-3p/FN1 might be necessary in the progresses of myocardial fibrosis in DCM.

To assess the strength of the miRNA-mRNA network analysis, we filtered 10 mRNAs and genes including miR-144-3p, miR-144-5p, miR-9-3p, miR-363-3p, miR-338-3p, miR-770-5p, miR-21-3p, ID4, PIK3R1, and FN1, which are believed to play critical roles in the development and pathological mechanisms of DCM. Among which, miR-9-3p and miR-144-3p were verified to be the most important functional miRNA, being recognized as the most important signaling pathways in DCM.

This study had some limitations. Firstly, the underlying mechanisms of DCM have to be further explained, calling for an establishment of the triplex network for genes, miRNAs, transcription factors, and mRNAs. Furthermore, due to the lack of experimental verification, these conclusions need to be further explored.

## 5. Conclusion

In this study, we constructed the miRNA-mRNA network in DCM pathogenesis and identified that the miR-144-3p/FN1 and miR-9-3p/FN1 pathways could be involved in the pathogenesis of myocardial fibrosis in DCM, providing potential therapeutic targets in DCM disease.

## Figures and Tables

**Table 1:** All nine differentially expressed miRNAs (DEMis) in dilated cardiomyopathy samples.

Name	Log FC	*P* value	Adjusted *P* value
miR-144-3p	-7.7311	<0.001	<0.001
miR-144-5p	-6.7954	<0.001	<0.001
miR-9-3p	-6.1527	<0.001	<0.001
miR-451a	-2.8682	<0.001	<0.001
miR-551b-3p	-2.3371	<0.001	<0.001
miR-363-3p	-1.7493	<0.001	<0.001
miR-338-3p	-1.7096	<0.001	<0.001
miR-770-5p	4.331	<0.001	<0.001
miR-21-3p	4.9264	<0.001	<0.001

**Table 2:** Top 40 differentially expressed mRNAs, half upregulated, half downregulated.

Name	Log FC	*P* value	Adjusted *P* value	Name	Log FC	*P* value	Adjusted *P* value
Top 20 upregulated mRNAs				Top 20 downregulated mRNAs			
NPPB	4.5	2.50*E*-06	6.58*E*-03	RARRES1	-2.07	1.15*E*-04	1.69*E*-02
NPPA	2.24	1.97*E*-05	9.24*E*-03	S100A8	-2.02	3.51*E*-04	3.00*E*-02
CTGF	1.92	8.89*E*-05	1.52*E*-02	CORIN	-1.84	8.04*E*-03	1.17*E*-01
CFH	1.76	3.63*E*-06	6.58*E*-03	CCL2	-1.48	9.68*E*-04	4.55*E*-02
FRZB	1.63	3.99*E*-04	3.11*E*-02	MYH6	-1.33	8.71*E*-03	1.22*E*-01
ACE2	1.39	8.37*E*-04	4.55*E*-02	FCN3	-1.27	7.57*E*-03	1.15*E*-01
SPOCK1	1.34	1.70*E*-04	2.02*E*-02	C1orf105	-1.22	2.28*E*-04	2.43*E*-02
AEBP1	1.32	5.76*E*-04	3.68*E*-02	ETNPPL	-1.21	2.03*E*-03	6.45*E*-02
ASPN	1.28	5.23*E*-04	3.44*E*-02	G0S2	-1.16	4.24*E*-04	3.16*E*-02
LTBP2	1.27	8.50*E*-04	4.55*E*-02	DLK1	-1.13	1.38*E*-02	1.48*E*-01
PHLDA1	1.25	7.49*E*-06	6.58*E*-03	B3GALT2	-1.07	3.83*E*-03	8.38*E*-02
SFRP4	1.25	9.66*E*-04	4.55*E*-02	CD14	-1.05	7.00*E*-03	1.13*E*-01
OGN	1.19	6.45*E*-03	1.10*E*-01	PPP1R1A	-1.05	1.33*E*-03	5.36*E*-02
ELN	1.19	1.31*E*-03	5.32*E*-02	LYVE1	-0.96	3.12*E*-03	7.63*E*-02
POSTN	1.18	1.42*E*-02	1.50*E*-01	F13A1	-0.94	1.47*E*-02	1.51*E*-01
ODC1	1.17	2.56*E*-06	6.58*E*-03	CCDC69	-0.93	1.53*E*-04	2.02*E*-02
RRAS2	1.15	2.52*E*-02	1.91*E*-01	PTP4A3	-0.91	2.39*E*-05	9.69*E*-03
COL1A1	1.13	4.30*E*-03	8.98*E*-02	NR4A3	-0.9	3.83*E*-02	2.29*E*-01
COL1A2	1.13	1.39*E*-03	5.54*E*-02	SELENBP1	-0.89	9.77*E*-05	1.64*E*-02
NAP1L3	1.13	3.43*E*-03	7.99*E*-02	SIK1	-0.88	3.67*E*-03	8.25*E*-02

**Table 3:** The top 10 nodes in the regulatory network.

Node name	Degree	Closeness	Betweenness
miR-144-3p	17	27.0	1196.7
miR-363-3p	15	24.5	799.3
miR-9-3p	14	24.35	741.1
miR-21-3p	7	7.0	42.0
miR-144-5p	4	16.23	178.9
miR-338-3p	4	14.78	258
ID4	3	20.67	257.9
miR-770-5p	3	3.0	6.0
PIK3R1	2	18.62	59.3
FN1	2	19.5	79.9

## Data Availability

No data were used to support this study.

## References

[1] Merlo M., Gigli M., Poli S. (2016). Dilated cardiomyopathy: a dynamic disease - clinical course, reverse remodeling and prognostic stratification. *Giornale Italiano Di Cardiologia*.

[2] Elliott P., Andersson B., Arbustini E. (2008). Classification of the cardiomyopathies: a position statement from the European Society of Cardiology working group on myocardial and pericardial diseases. *European Heart Journal*.

[3] Calderon-Dominguez M., Belmonte T., Quezada-Feijoo M. (2020). Emerging role of microRNAs in dilated cardiomyopathy: evidence regarding etiology. *Translational Research*.

[4] Esteller M. (2011). Non-coding RNAs in human disease. *Nature Reviews. Genetics*.

[5] Rottiers V., Naar A. M. (2012). MicroRNAs in metabolism and metabolic disorders. *Nature Reviews. Molecular Cell Biology*.

[6] Tao L., Yang L., Huang X., Hua F., Yang X. (2019). Reconstruction and analysis of the lncRNA-miRNA-mRNA network based on competitive endogenous RNA reveal functional lncRNAs in dilated cardiomyopathy. *Frontiers in Genetics*.

[7] Gioffré S., Ricci V., Vavassori C. (2019). Plasmatic and chamber-specific modulation of cardiac microRNAs in an acute model of DOX-induced cardiotoxicity. *Biomedicine & Pharmacotherapy*.

[8] Chen L., Hou X., Zhang M. (2020). MicroRNA-223-3p modulates dendritic cell function and ameliorates experimental autoimmune myocarditis by targeting the NLRP3 inflammasome. *Molecular Immunology*.

[9] Toro R., Blasco-Turrión S., Morales-Ponce F. J. (2018). Plasma microRNAs as biomarkers for Lamin A/C-related dilated cardiomyopathy. *Journal of Molecular Medicine*.

[10] Wu T., Chen Y., du Y., Tao J., Zhou Z., Yang Z. (2018). Serum exosomal miR-92b-5p as a potential biomarker for acute heart failure caused by dilated cardiomyopathy. *Cellular Physiology and Biochemistry*.

[11] Rubiś P., Totoń-Żurańska J., Wiśniowska-Śmiałek S. (2018). The relationship between myocardial fibrosis and myocardial microRNAs in dilated cardiomyopathy: a link between mir-133a and cardiovascular events. *Journal of Cellular and Molecular Medicine*.

[12] Zhou Q., Schötterl S., Backes D. (2017). Inhibition of miR-208b improves cardiac function in titin-based dilated cardiomyopathy. *International Journal of Cardiology*.

[13] Verjans R., Peters T., Beaumont F. J. (2018). MicroRNA-221/222 family counteracts myocardial fibrosis in pressure overload-induced heart failure. *Hypertension*.

[14] Ebert M. S., Sharp P. A. (2010). MicroRNA sponges: progress and possibilities. *RNA*.

[15] Barth A. S., Kuner R., Buness A. (2006). Identification of a common gene expression signature in dilated cardiomyopathy across independent microarray studies. *Journal of the American College of Cardiology*.

[16] Zou J. B., Chai H. B., Zhang X. F. (2019). Reconstruction of the lncRNA-miRNA-mRNA network based on competitive endogenous RNA reveal functional lncRNAs in cerebral infarction. *Scientific Reports*.

[17] Pathan M., Keerthikumar S., Ang C. S. (2015). FunRich: an open access standalone functional enrichment and interaction network analysis tool. *Proteomics*.

[18] Pathan M., Keerthikumar S., Chisanga D. (2017). A novel community driven software for functional enrichment analysis of extracellular vesicles data. *Journal of Extracellular Vesicles*.

[19] Zhou J., Hui X., Mao Y., Fan L. (2019). Identification of novel genes associated with a poor prognosis in pancreatic ductal adenocarcinoma via a bioinformatics analysis. *Bioscience Reports*.

[20] Hou Y., Wang Y., Xu S., Qi G., Wu X. (2019). Bioinformatics identification of microRNAs involved in polycystic ovary syndrome based on microarray data. *Molecular Medicine Reports*.

[21] Zhang H., Yu Z., He J., Hua B., Zhang G. (2017). Identification of the molecular mechanisms underlying dilated cardiomyopathy via bioinformatic analysis of gene expression profiles. *Experimental and Therapeutic Medicine*.

[22] Moreau A., Gosselin-Badaroudine P., Mercier A., Burger B., Keller D. I., Chahine M. (2018). A leaky voltage sensor domain of cardiac sodium channels causes arrhythmias associated with dilated cardiomyopathy. *Scientific Reports*.

[23] Orenes-Piñero E., Montoro-García S., Patel J. V., Valdés M., Marín F., Lip G. Y. H. (2013). Role of microRNAs in cardiac remodelling: new insights and future perspectives. *International Journal of Cardiology*.

[24] Besler C., Urban D., Watzka S. (2016). Endomyocardial miR-133a levels correlate with myocardial inflammation, improved left ventricular function, and clinical outcome in patients with inflammatory cardiomyopathy. *European Journal of Heart Failure*.

[25] Duisters R. F., Tijsen A. J., Schroen B. (2009). miR-133 and miR-30 regulate connective tissue growth factor: implications for a role of microRNAs in myocardial matrix remodeling. *Circulation Research*.

[26] Zhang X., Wang X., Zhu H. (2010). Synergistic effects of the GATA-4-mediated miR-144/451 cluster in protection against simulated ischemia/reperfusion-induced cardiomyocyte death. *Journal of Molecular and Cellular Cardiology*.

[27] Yuan X., Pan J., Wen L. (2019). miR-144-3p enhances cardiac fibrosis after myocardial infarction by targeting PTEN. *Frontiers in Cell and Development Biology*.

[28] Zheng J., Peng B., Zhang Y., Ai F., Hu X. (2019). miR-9 knockdown inhibits hypoxia-induced cardiomyocyte apoptosis by targeting Yap1. *Life Sciences*.

[29] Xiao Y., Zhang Y., Chen Y. (2019). Inhibition of microRNA-9-5p protects against cardiac remodeling following myocardial infarction in mice. *Human Gene Therapy*.

[30] Jin X., Yu L. L., Yu C. X. (2019). Effect of miR-9 on myocardial fibrosis in rats via TGF-*β*1/Smads signaling pathway. *European Review for Medical and Pharmacological Sciences*.

[31] Meng X., Ji Y., Wan Z. (2017). Inhibition of miR-363 protects cardiomyocytes against hypoxia-induced apoptosis through regulation of Notch signaling. *Biomedicine & Pharmacotherapy*.

[32] Li K., Lin Y., Li C. (2019). miR-338-5p ameliorates pathological cardiac hypertrophy by targeting CAMKII*δ*. *Archives of Pharmacal Research*.

[33] Zhao H., Yu X., Ding Y. (2016). miR-770-5p inhibits cisplatin chemoresistance in human ovarian cancer by targeting ERCC2. *Oncotarget*.

[34] Sun B., Liu H. F., Ding Y., Li Z. (2018). Evaluating the diagnostic and prognostic value of serum miR-770 in non-small cell lung cancer. *European Review for Medical and Pharmacological Sciences*.

[35] Yang L., Wang B., Zhou Q. (2018). MicroRNA-21 prevents excessive inflammation and cardiac dysfunction after myocardial infarction through targeting KBTBD7. *Cell Death & Disease*.

[36] Wei C., Li L., Kim I. K., Sun P., Gupta S. (2013). NF-*κ*B mediated miR-21 regulation in cardiomyocytes apoptosis under oxidative stress. *Free Radical Research*.

[37] Wang J., Duan L., Gao Y. (2018). Angiotensin II receptor blocker valsartan ameliorates cardiac fibrosis partly by inhibiting miR-21 expression in diabetic nephropathy mice. *Molecular and Cellular Endocrinology*.

[38] Young M. E., Brewer R. A., Peliciari-Garcia R. A. (2014). Cardiomyocyte-specific BMAL1 plays critical roles in metabolism, signaling, and maintenance of contractile function of the heart. *Journal of Biological Rhythms*.

[39] Chen Y., Zhu D., Yuan J. (2016). CLOCK-BMAL1 regulate the cardiac L-type calcium channel subunit CACNA1C through PI3K-Akt signaling pathway. *Canadian Journal of Physiology and Pharmacology*.

[40] Duffield G. E., Watson N. P., Mantani A. (2009). A role for Id2 in regulating photic entrainment of the mammalian circadian system. *Current Biology*.

[41] Kang S., Verma S., Hassanabad A. F. (2020). Direct effects of empagliflozin on extracellular matrix remodelling in human cardiac myofibroblasts: novel translational clues to explain EMPA-REG OUTCOME results. *The Canadian Journal of Cardiology*.

